# Higher incidence of zinc and nickel hypersensitivity in patients with irritable bowel syndrome

**DOI:** 10.1002/iid3.274

**Published:** 2019-10-24

**Authors:** Yasunari Kageyama, Koichi Aida, Kimihiko Kawauchi, Masafumi Morimoto, Tetsu Akiyama, Tsutomu Nakamura

**Affiliations:** ^1^ Takanawa Clinic Tokyo Japan; ^2^ Laboratory of Molecular and Genetic Information Institute for Quantitative Biosciences, The University of Tokyo Tokyo Japan

**Keywords:** hypersensitivity, irritable bowel syndrome, nickel, zinc

## Abstract

**Introduction:**

The etiology of irritable bowel syndrome (IBS) remains elusive even though several genetic and environmental pathogenic factors have been reported. IBS is considered to be a functional disorder without any detectable lesions in the patient's bowel. However, many studies have demonstrated that a subset of IBS patients have low‐grade inflammation and aberrant T‐cell activation in their intestinal mucosa. To elucidate the immune mechanism underlying the mucosal inflammation in IBS, we focused on dental metal hypersensitivity, a T cell–mediated, delayed‐type allergic reaction that causes oral contact mucositis and systemic cutaneous inflammation.

**Methods:**

We recruited 147 Japanese IBS patients and 22 healthy controls (HCs). The subjects underwent the in vitro lymphocyte stimulation test to quantify their sensitivity to zinc, gold, nickel, and palladium, the metals that have been commonly used in dentistry.

**Results:**

A total of 56.5% of the IBS patients were hypersensitive to at least one metal species, whereas 31.8% of HC were hypersensitive to only a single metal species. The overall incidence of metal hypersensitivity was significantly higher for IBS patients than for HC. Furthermore, a significantly higher proportion of IBS patients were hypersensitive to zinc and/or nickel. The severity of the sensitivity to zinc and nickel was also significantly greater for IBS patients than for HC. There was no significant difference in the sensitization rates and the sensitivity among the IBS subtypes.

**Conclusions:**

This pilot study demonstrates that IBS patients have a significantly higher prevalence of hypersensitivity to zinc and nickel, suggesting the possible involvement of dental metal hypersensitivity in IBS pathogenesis in a subset of patients.

Irritable bowel syndrome (IBS) is a chronic intestinal condition with a global prevalence of ~10% and is characterized by recurrent abdominal symptoms such as crampy pain, bloating, constipation, and/or diarrhea. The etiology is not fully understood even though several contributing factors have been proposed, including social stress, dysregulated gut‐brain axis, bacterial or viral infection, gut dysbiosis, and genetic predisposition. IBS is considered to be a functional disorder of the bowel without any detectable structural lesions. However, many studies have demonstrated the presence of low‐grade mucosal inflammation and aberrant T cell activation at the microscopic and molecular levels in a subset of IBS patients.

To elucidate the immune mechanism underlying IBS, we focused on hypersensitivity to dental metals, which involves a T cell‐mediated, delayed‐type allergic reaction.^1^ The condition is characterized by oral contact mucositis and systemic skin inflammation that has a close etiological relationship with palmoplantar pustulosis, lichen planus, and dyshidrotic eczema.^2^ Notably, metal hypersensitivity is also closely associated with certain chronic inflammatory autoimmune diseases that include systemic lupus erythematosus, rheumatoid arthritis, and Sjögren's syndrome.^3^ We hypothesized that persistent release of trace amounts of the metal cations from dental alloys could provoke the chronic, low‐grade inflammation observed in the IBS intestinal mucosa. We thus conducted a case‐control study to explore the incidence of hypersensitivity to zinc, gold, nickel, and palladium that have been commonly used for dental prostheses.^4^

A total of 147 Japanese patients and 22 healthy controls (HC) were enrolled. Each patient was diagnosed with IBS at the gastroenterology department of Takanawa Clinic from October 2017 through July 2018. The patients were classified into four subtypes according to the Rome IV diagnostic criteria: 59 diarrhea‐predominant IBS (IBS‐D; 40.1%), 9 constipation‐predominant IBS (IBS‐C; 6.1%), 66 IBS with mixed bowel habits (IBS‐M; 44.9%), and 13 unspecified IBS (IBS‐U; 8.8%). The drug‐induced lymphocyte stimulation test (DLST, also termed lymphocyte transformation test), an in vitro method for detecting metal sensitization quantitatively,^5^ was outsourced to SRL, Inc (Tokyo, Japan). Any subject with a stimulation index (SI) of 180 or more was regarded as sensitization‐positive. Details of the subjects and experimental procedures are available in the Supporting Information.

The subjects underwent the DLST for zinc, gold, nickel, and palladium. For HC, the majority of the subjects (68.2%) were negative for sensitization to any metals examined, and all the rest of the HC individuals (31.8%) were positive for sensitization to only a single metal species (Figure 1A). In contrast, more than half (56.5%) of IBS patients were sensitized to at least one metal species, and a considerable subset of those patients (25.9%) had hypersensitivity to more than one metal species. Two IBS patients were hypersensitive to all four metal species examined. Notably, the overall incidence of metal sensitization was significantly higher for IBS patients compared with HC (*P* = .0392, two‐tailed Fisher's exact test; the odds ratio = 2.78, 95% confidence interval: 1.07‐7.22; Figure 1A).

**Figure 1:**
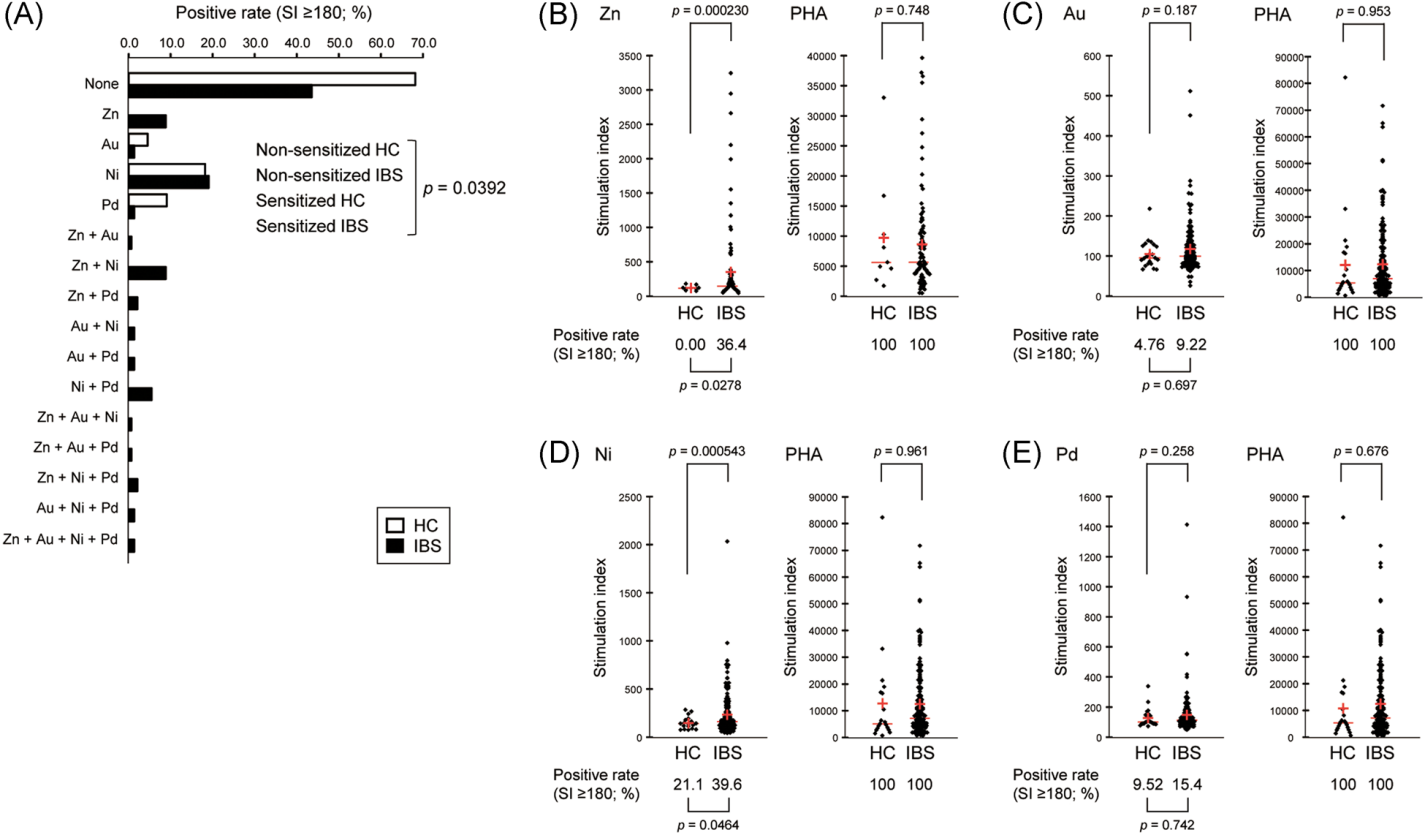
IBS patients are more sensitive to zinc and nickel than are healthy individuals. A, The incidence of hypersensitivity to zinc, gold, nickel, and/or palladium (SI ≥ 180) in HCs (open bar) and the patients (IBS, closed bar). B‐E, Distribution of SI values for HCs and patients (IBS) with respect to zinc (B), gold (C), nickel (D), and palladium (E). Each red horizontal line and red cross correspond to the median and the mean, respectively. DLST‐positive rates (SI ≥ 180) are shown below the beeswarms. PHA was used as a positive control in each test. The data were statistically analyzed with two‐tailed Fisher's exact test (A‐E) and two‐tailed Welch's *t* test (B–E). DLST, drug‐induced lymphocyte stimulation test; HC, healthy control; IBS, irritable bowel syndrome; PHA, phytohemagglutinin; SI, stimulation index [Color figure can be viewed at wileyonlinelibrary.com]

In support of this result, a significantly higher proportion of IBS patients were hypersensitive to zinc (36.4% [IBS] vs 0.00% [HC]; *P* = .0278, two‐tailed Fisher's exact test; Figure 1B) or nickel (39.6% [IBS] vs 21.1% [HC]; *P* = .0464, two‐tailed Fisher's exact test; Figure 1D). Similarly, mean SI values were significantly higher for IBS than HC with respect to both zinc (352 [IBS] vs 119 [HC]; *P* = .000230, two‐tailed Welch's *t* test; Figure 1B) and nickel (233 [IBS] vs 147 [HC]; *P* = .000543, two‐tailed Welch's *t* test, Figure 1D). However, the incidence of individuals who were hypersensitive to gold or palladium, as well as the corresponding mean SI values, were relatively low, without any significant difference between IBS and HC (Figure 1C and 1E).

We compared sensitization rates to zinc and nickel and the SI values among the four IBS subtypes, but there was no significant difference (sensitization rate: *P* = 0.216 [zinc], *P* = .989 [nickel], two‐tailed Fisher's exact test; SI value: *P* = .535 [zinc], *P* = .495 [nickel], one‐way analysis of variance; Figure 2).

**Figure 2:**
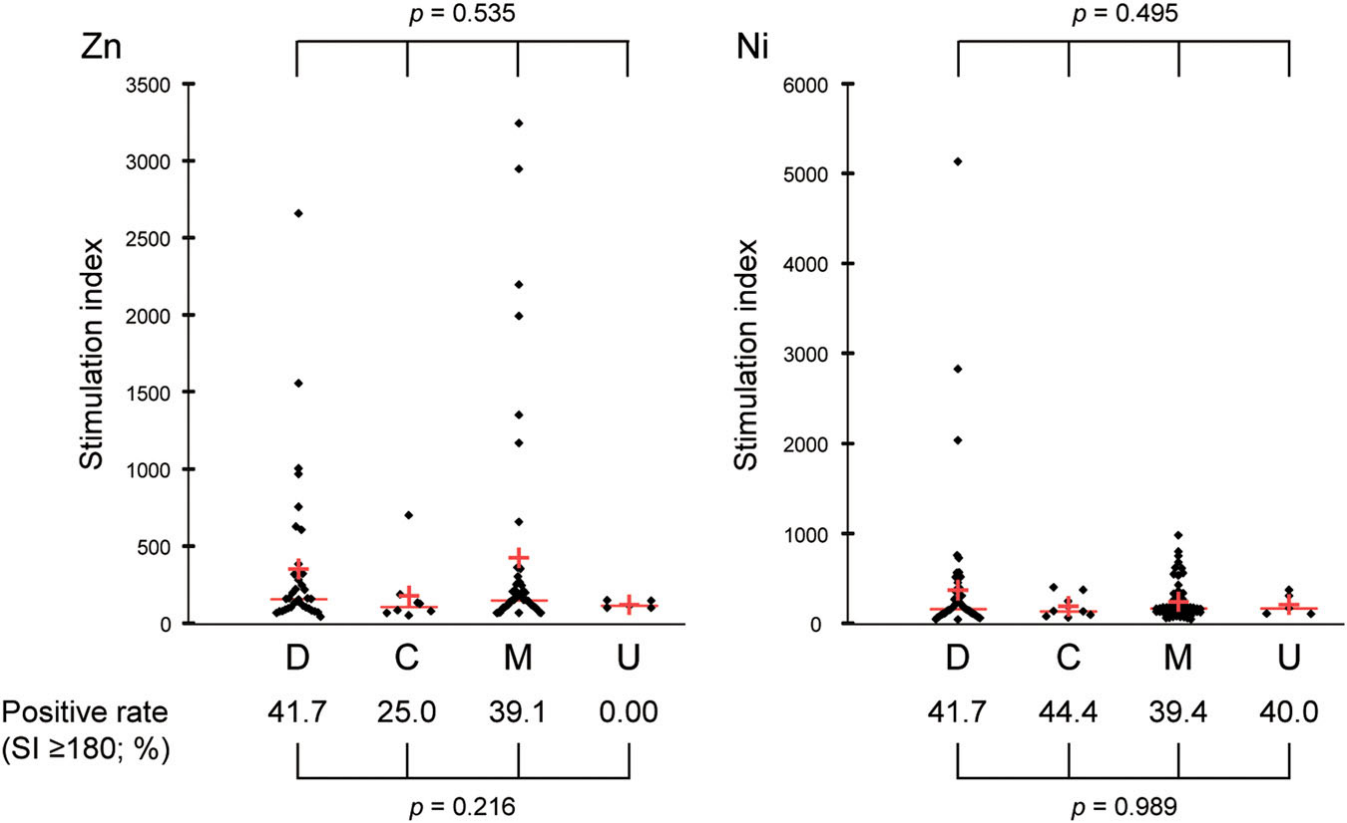
Comparison of sensitization rates and SI values among IBS subtypes. The data for zinc (left) and nickel (right) DLST are shown by IBS subtype. Each red horizontal line and red cross correspond to the median and the mean, respectively. The incidence of sensitization to zinc or nickel (SI ≥ 180) in each IBS subtype is shown below the beeswarms. D, IBS‐D; C, IBS‐C; M, IBS‐M; and U, IBS‐U. The data were statistically analyzed with one‐way analysis of variance and two‐tailed Fisher's exact test. DLST, drug‐induced lymphocyte stimulation test; IBS, irritable bowel syndrome; IBS‐C, constipation‐predominant IBS; IBS‐D, diarrhea‐predominant IBS; IBS‐M, IBS with mixed bowel habits; IBS‐U, unspecified IBS; SI, stimulation index [Color figure can be viewed at wileyonlinelibrary.com]

There is increasing recognition that the mucosal immune response mediated by CD4^+^ and CD8^+^ T cells plays an important role in the bowel symptoms of IBS.^6^ The numbers of mucosal CD3^+^, CD4^+^, and CD8^+^ T cells and mast cells and mucosal infiltration by CD4^+^ T cells are increased in colonic biopsies from a large subset of IBS patients. Furthermore, activated, gut‐homing CD4^+^ and CD8^+^ T cells are increased in the peripheral blood of IBS patients. Mucosal CD4^+^ T cells and mast cells from IBS patients are known to stimulate visceral nociceptive sensory neurons, suggesting that activation of mucosal immune cells in IBS patients leads to visceral hypersensitivity and altered colon motility.

Similar T cell activation is known to accompany zinc and nickel hypersensitivity.^7^ Zinc hypersensitivity elicits a lichenoid tissue reaction that involves the subepidermal infiltration by CD8^+^ T cells. The treatment of dendritic cells with nickel promotes the expansion of IL‐17A‐producing CD4^+^ T cells that play critical roles in the pathogenesis of inflammatory bowel disease. The incidence of individuals with nickel hypersensitivity is significantly higher in patients with nonceliac wheat sensitivity, a gluten‐related disease characterized by a combination of IBS‐like bowel symptoms. Furthermore, the majority of IBS patients report that their bowel symptoms are triggered by specific foods or food components; notably, a considerable subset of them are particularly rich in zinc and nickel.^8^ Rizzi et al^9^ demonstrated that a low‐nickel diet could clearly ameliorate the bowel symptoms of IBS patients. Thus, chronic exposure to zinc and nickel cations derived from dental alloys may provoke IBS symptoms through mucosal T cell activation.

This pilot study suggests that hypersensitivity reaction to orally derived zinc and nickel may underlie aberrant immune activation in the IBS mucosa; however, the limited number of subjects may limit its conclusions and generalizability. Further studies with larger cohorts and from mechanistic perspective will be needed to firmly establish the causative link between hypersensitivity to intraoral metals and IBS pathogenesis.

## CONFLICT OF INTERESTS

Yasunari Kageyama, Koichi Aida, Kimihiko Kawauchi, and Masafumi Morimoto are employees of Takanawa Clinic. Tetsu Akiyama and Tsutomu Nakamura have advisory roles in conducting clinical research in Takanawa Clinic and receive advisory fees from Takanawa Clinic.

## AUTHOR CONTRIBUTIONS

YK, KA, KK, and MM contributed to study concept and design, acquisition of data, analysis and interpretation of data, and critical revision of the manuscript for important intellectual content. TA contributed to study concept and design, critical revision of the manuscript for important intellectual content, and study supervision. TN contributed to study concept and design, analysis and interpretation of data, drafting of the manuscript, statistical analysis, and study supervision. All authors gave final approval and agree to be accountable for all aspects of the work.

## Supporting information

Supplementary information

## Data Availability

The data that support the findings of this study are available on request from the corresponding author. The data are not publicly available due to privacy or ethical restrictions.
