# Supplementary material for: Higher incidence of zinc and nickel hypersensitivity in patients with irritable bowel syndrome
Source: Immun Inflamm Dis. 2019 Oct 24;7(4):304–7. doi: 10.1002/iid3.274 (PMC6842818; doi:10.1002/iid3.274)
Supplement: Supplementary file 1 — Supplementary information [file IID3-7-304-s001.docx]

**Supporting Information**

**Subjects**

This study was carried out in accordance with The Code of Ethics of the World Medical Association (Declaration of Helsinki). All procedures were approved by the Ethics Committees of the Takanawa Clinic (the Ethics Committee approval number: 2018-2). A signed informed consent form was obtained from each participant prior to inclusion in this study. All information on the subjects was kept in a secure location, and data were analyzed using a de-identified dataset. All experimental procedures were conducted by investigators who were blinded to the clinical information of the subjects.

A total of 147 Japanese patients were enrolled (61 males and 86 females; age range 11–84 years; mean [SD] age, 40.2 [17.0] years). Each patient was diagnosed with IBS at the gastroenterology outpatient department of Takanawa Clinic from October 2017 through July 2018. The patients were classified into four subtypes according to the Rome IV diagnostic criteria for IBS: 59 diarrhea-predominant IBS (IBS-D; 40.1%), 9 constipation-predominant IBS (IBS-C; 6.1%), 66 IBS with mixed bowel habits (IBS-M; 44.9%), and 13 unspecified IBS (IBS-U; 8.8%). Patients who were over 85 years old or who were pregnant were excluded. A total of 22 healthy controls (HC) were recruited (7 males and 15 females; ages 12–70 years; mean [SD] age, 48.9 [13.7] years). Individuals were excluded from the HC group if they had current or previous IBS or other major GI diseases. Individuals with inflammatory diseases and hypersensitivity to allergen(s) other than metals were also excluded from both HC and IBS groups.

**DLST**

The drug-induced lymphocyte stimulation test (DLST, also termed lymphocyte transformation test or LTT), an *in vitro* method for detecting metal sensitization quantitatively, was outsourced to SRL, Inc. (Tokyo, Japan). Briefly, lymphocytes were isolated from heparinized peripheral blood by density gradient centrifugation using Ficoll-Conray media (Lymphosepar I, Immuno-Biological Laboratories) and resuspended at 1 × 10^6^/ml in RPMI1640 medium supplemented with 20% autologous plasma. The 200 μl aliquots (2 × 10^5^ cells) were cultured in triplicate in a 96-well microplate with metal cations or phytohemagglutinin (PHA; 7 μg/ml) at 37°C for 72 hours in a 5% CO_2_ incubator. Proliferation activity was measured by the addition of [Methyl-^3^H]thymidine (10.57 kBq/well, Moravek Inc.) for 18 hours, followed by harvesting of cells on filters. Radioactivity retained on the filters was measured using a scintillation counter (PerkinElmer Inc.). Any subject with a stimulation index (SI) of 180 or more was regarded as sensitization-positive: SI = 100 × [the amount (cpm) of ^3^H-thymidine incorporated into peripheral blood lymphocytes of a subject during stimulation with a metal species / the amount (cpm) with no stimulation]. Phytohemagglutinin (PHA) was used as a positive control.

**Statistical analysis**

Statistical analyses were performed using Prism8 software (GraphPad Software, San Diego, CA, USA). No outliers were taken into account, and all collected data were subjected to statistical analysis. Two-tailed Fisher's exact test was used to evaluate the significance of the distribution difference between IBS patients and HC and among IBS subtypes. Statistical significance of the difference between two mean values was tested using two-tailed Welch’s *t*-test or one-way analysis of variance (ANOVA). *P*-values of less than 0.05 were regarded as statistically significant.
